# Oral Implant-Prostheses: New Teeth for a Brighter Brain

**DOI:** 10.1371/journal.pone.0148715

**Published:** 2016-02-26

**Authors:** Vincenzo De Cicco, Massimo Barresi, Maria Paola Tramonti Fantozzi, Enrico Cataldo, Vincenzo Parisi, Diego Manzoni

**Affiliations:** 1 Department of Translational Research, University of Pisa, Pisa, Italy; 2 Department of Drug Sciences, University of Catania, Catania, Italy; 3 Department of Physics, University of Pisa, Pisa, Italy; 4 GB Bietti Foundation, IRCCS, Roma, Italy; Duke University, UNITED STATES

## Abstract

Several studies have demonstrated that chewing can be regarded as a preventive measure for cognitive impairment, whereas masticatory deficiency, associated with soft-diet feeding, is a risk factor for the development of dementia. At present the link between orofacial sensorimotor activity and cognitive functions is unknown. In subjects with unilateral molar loss we have shown asymmetries in both pupil size and masticatory muscles electromyographic (EMG) activity during clenching: the molar less side was characterized by a lower EMG activity and a smaller pupil. Since implant-prostheses, greatly reduced both the asymmetry in EMG activity and in pupil’s size, trigeminal unbalance, leading to unbalance in the activity of the Locus Coeruleus (LC), may be responsible for the pupil’s asymmetry. According to the findings obtained in animal models, we propose that the different activity of the right and left LC may induce an asymmetry in brain activity, thus leading to cognitive impairment. According to this hypothesis, prostheses improved the performance in a complex sensorimotor task and increased the mydriasis associated with haptic tasks. In conclusion, the present study indicates that the implant-prosthesis therapy, which reduces the unbalance of trigeminal proprioceptive afferents and the asymmetry in pupil’s size, may improve arousal, boosting performance in a complex sensorimotor task.

## Introduction

Previous studies reported that mastication improves cognitive processing speed [1], alertness [2], attention [3], intelligence [4], as well as reaction time [5,6], event-related potentials latencies [7] and cerebral blood oxygen-dependent (Bold) signal [6]. It has been proposed that chewing may enhances arousal and modulate cognitive functions [7] by enhancing the activity of Ascending Reticular Activating System [8]. In additions to these short-term effects on performance, it has been suggested that the cerebral cortex activity elicited by mastication may lead to long term effects on the cerebral nervous system and be helpful in preventing degradation of brain functions [9,10,11]. Indeed, epidemiological studies have reported that tooth loss before 35 years of age was a significant risk factor for dementia or Alzheimer Disease [12,13]. In animal experiments, it has been well documented that tooth loss, leading to long-term masticatory unbalance, decreases the number of pyramidal cells in the Hippocampal CA1 and Gyrus Dentatus [14] with impairment of spatial learning and memory in water maze tests [15]. These deficits seem to increase with aging, soft-diet feeding and time after tooth loss [16,17]. Tooth loss also increases the proliferation and the hypertrophy of hippocampal astrocytes, as it occurs following neuronal degeneration and senescence processes [16]; moreover, at hippocampal level, it decreases the number of neurons expressing c-Fos during spatial task [18], the number of dendritic spines [19] and neurogenesis [20]. It is noteworthy that the reduction in the number of c-Fos-positive cells in the hippocampal CA1 region was partially antagonized by restoring the lost molars with artificial crowns [21]. Other studies on molar less mice showed plasma glucocorticoid levels significantly greater than in molar intact control mice [22] and it is known that glucocorticoids may lead to suppression of synaptic plasticity in hippocampal neurons [23]. In addition to tooth loss, also a soft diet may affect brain structures, leading to reduced levels of brain-derived neurotrophic factor (BDNF) [24] and hippocampal neurogenesis [25]. So, there is a huge evidence that chronic masticatory dysfunction may affect brain neurobiology.

Recent studies on short-term effects of masticatory deficits on brain activity have shown that patients with temporo-mandibular disorders (TMD) show an asymmetry in both pupil size and electromyographic (EMG) activity of masticatory muscles during clenching. Moreover, the reduction of the former asymmetry by occlusal correction greatly reduce the latter, and enhances the mydriatic response associated with haptic task [26]. It is known that task-related mydriasis reflects task-associated “arousal” [27,28], “mental effort” [29,30] and, maybe, task performance (see [31]). These findings indicates that 1) trigeminal sensorimotor activity exerts a tonic effect on autonomic structures controlling pupil size and 2) its unbalance impairs cognitive performance, which is in line with the trigeminal role in long-term neurodegenerative processes shown in animal experiments and clinical studies [9–25].

However, a deeper insight is still required into the effects of occlusal unbalance and its correction upon brain activity and subject’s performance. Thus the purpose of the present experiment was to investigate whether subjects with unilateral molar loss 1) show an asymmetry in the EMG activity of masticatory muscles and pupil diameter and whether replacement of the lost teeth by implant-supported prostheses 2) decrease asymmetry, 3) increases the task associated arousal estimated by the recording of mydriatic response during a haptic task, and 4) increase the sensorimotor/cognitive performance (assessed by the Spinnler-Tognoni numeric matrices [32]).

## Methods

### Subjects

Nine subjects (5 males and 4 female; age (mean ± SD) 46.4 ± 7.7 years) were enrolled in the present study. They showed an unilateral loss of the first and second inferior molars, either on the left (n = 4) or on the right side (n = 5) and underwent implant of dental prostheses for restoring normal occlusal surface.

In order to evaluate the effects of simple test repetition on performance, we also studied a population of nine subjects (3 males and 6 females; age 40.2 ± 12.1 years), without occlusal alterations (controls).

Experiments consisted in routine dental care interventions, which were part of the professional practice of one of the authors (VDC) and were aimed at correcting occlusal alterations in patients and performing a preventive screening for possible deficits in normal subjects. All the subjects signed an informed consent describing the experimental design and agreed to participate in a post-operative follow-up. They were asked to avoid caffeine and smoking for at least 2 hours before testing. None of the subjects was affected by bruxism, pain to masticatory/neck muscles, neurological, psychiatric, metabolic or endocrine diseases. None of them was under beta-blockers or corticosteroids therapy.

### Surgery and implants

After radiographic bone examinations, two or three one-piece implants (3P Implafavourite, Torino, Italy), were inserted to replace the first and second molar of the mandibular arch on the left (n = 4) or the right side (n = 5). The inserted implants (n = 22) were made by a single block and screwed into a hole drilled into the bone without preliminary crest incision, piercing directly the gums. Their dimensions (diameter/length) corresponded to 4.5/10 mm (n = 10), 4.5/12 mm (n = 4) or 5.2/10 mm (n = 8). Preliminary local anaesthesia was induced by infiltration with articaine/epinephrine (Pierrel, Italy, 1/100000, 2cc). Soon after the implant placement, dental impressions were taken so to manufacture the artificial prostheses (crowns) to be mounted on the implants. The occlusal contacts of prostheses with antagonist teeth were circumscribed to dental vestibular cusps. An antimicrobial prophylaxis (Amoxycillin, Pfizer, Italy, 500 mg, twice daily) was administered for 3 days, starting 1 hour before surgery. Following the surgery, analgesic (Nimesulide, SANDOZ S.P.A, Italy, 100 mg, twice daily) was delivered for 2 days.

### Experimental design

**Fig 1:**
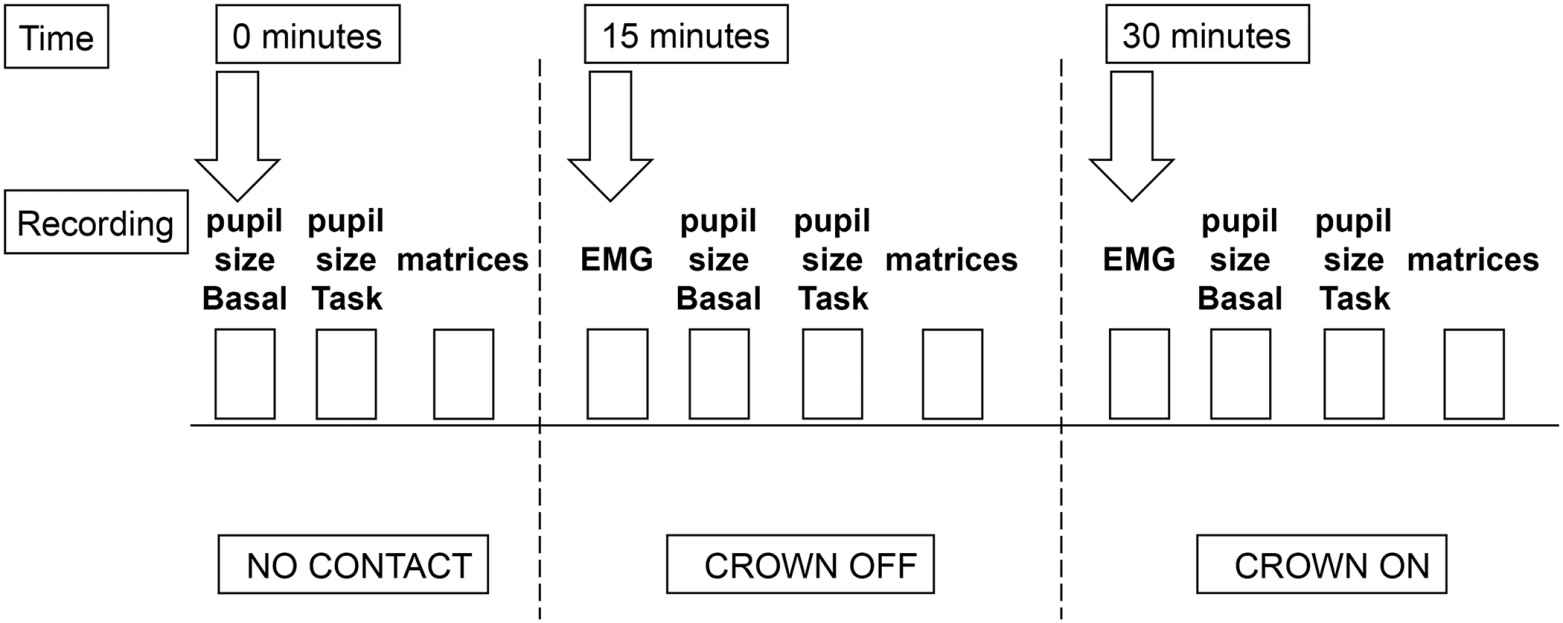
Experimental protocol. Flow diagram of the tests performed in all the patients at the different times. Pupil size was evaluated while the subjects were not engaged in any activity (Basal) and when they were performing a haptic task (Task). NO CONTACT: arches 1–2 mm apart. CROWN OFF: arches touching each other, no crowns on the implants. CROWN ON: arches touching each other, crowns inserted on the implants. See text for further explanation.

**Fig 2:**
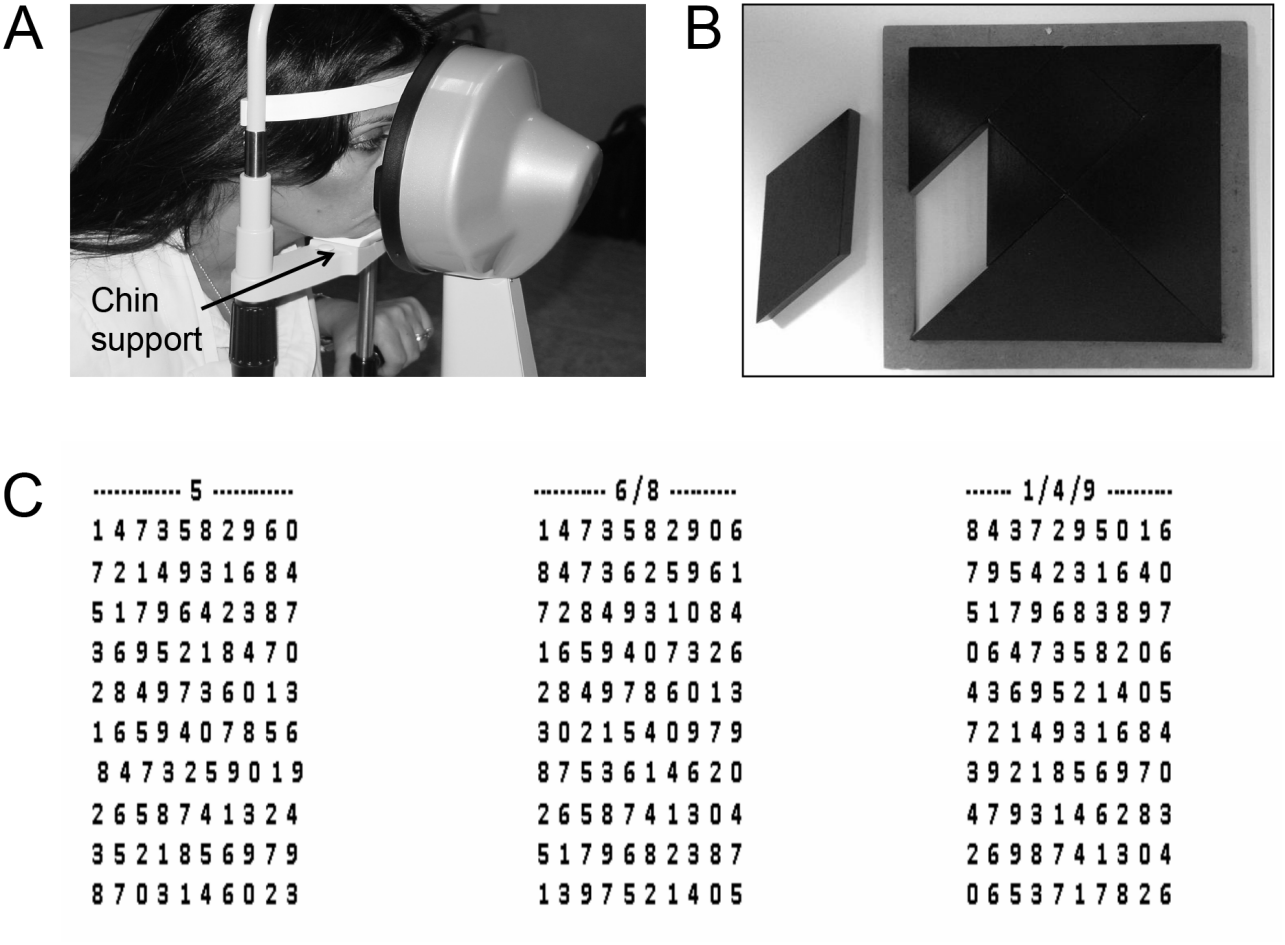
Pupil size recording and haptic task. A. Photograph of a representative subject with the head restrained in the pupillometric device. Note the bar for chin support that was lowered when recordings were taken with the arches 1–2 mm apart. B. Tan Gram puzzle. The parallelogram was put in the hand of the subject, while his/her head was restrained by the pupillometer as in A. The subject had to haptically reposition the piece within the puzzle. C. Example of Spinnler-Tognoni matrices. The subjects had to retrieve, from each matrix, the numbers indicated above it, underlying them with a pencil.

Fifteen days after surgery, temporary crowns were placed on the implants and occlusal condition was examined, in order to correct the prostheses appropriately. Then, 15 days later, before positioning of the temporary prostheses, subjects underwent evaluation of the following parameters with dental arches not touching each other (NO CONTACT condition) (see Fig 1):

1. basal pupils size evaluation while the subjects were not involved in any activity (Fig 2A);
2. pupils size evaluation during performance of a haptic task (Tan Gram) (Fig 2B),
3. retrieval of instructed digits from Spinnler-Tognoni numeric matrices (Fig 2C). The velocity (number/sec) of number retrieved was indicated as Performance Index (PI);

Tests 1–3 were repeated with dental arches in contact (CROWN OFF condition). In this condition 4) EMG recordings of both left and right masseter activity during a clenching effort were also performed.

At this point crowns were positioned without dental cement and tests 1–4 repeated once more with dental arches in contact (CROWN ON condition).

Five of the nine subjects enrolled in the experiment (2 males and 3 females; age 47.4 ± 7.5 years) were re-tested six months following the initial session. In this second session subjects wore the final prostheses, fixed with temporary cement. Pupil size was measured and the Tan Gram and Spinnler-Tognoni numeric matrices were performed in different consecutive occlusal condition: NO CONTACT, CROWN OFF (1), CROWN ON, CROWN OFF (2). EMG evaluations were performed only in CROWN ON and CROWN OFF. With respect to the initial session, the CROWN OFF (2) condition allowed to better distinguish the effects of occlusal condition from those of test repetition.

To this aim, we performed another experiment in control of subjects showing an asymmetric EMG activity of elevator muscles during clenching, but without any occlusal alteration. They were tested for three successive times in the NO CONTACT position.

### Pupil size evaluation

Pupil size measurements (mm) were performed in standard condition of artificial lighting by using a corneal topographer-pupillographer (MOD i02, with chin support, CSO, Florence, Italy) made up of a standard illuminator (halogen lamp, white light, ensuring a constant luminance level) and a camera sensor CCD1/3”, with a 56 mm working distance. The operator monitored the iris image by the camera, which had an acquisition time of 33 msec. Measurements were performed for both eyes in photopic conditions, (40 lux). The arches could be 1) in contact, the chin being supported (Fig 2A) and 2) 1–2 mm apart, without chin support. Diameter values were displayed online on the computer screen. During pupil size monitoring, the subjects did not perform any clenching effort. Pupil’s size was evaluated with the subject still and during the performance of a haptic task, which was practiced early only once, at the beginning of the experimental session. The task (Tan Gram), consisted of a puzzle of triangular, square and parallelogram-shaped forms. A piece of the puzzle (the parallelogram) (Fig 2B) was removed by the experimenter and put in the right hand of the subject, who had to fit it back in the original place without looking at his/her hand, keeping the head placed into the pupillometer. When the subject was at the rest, measurements were taken twice and only the second camera shot was utilised, while during task only one shot was taken, as soon as the subject began to explore the puzzle surface.

### Numeric Matrix Test

In the Spinnler-Tognoni matrices test the subjects seated in front of a table, where the operator discovered a leaf containing three numerical matrices, of 10 line and 10 columns. The subjects had been previously instructed to retrieve the number 5 from the first matrix from the left, the numbers 6 and 8 from the second and the numbers 1, 4 and 9 from the third, by underlying them with a pencil (see Fig 2C). The target numbers were on the whole 60 out of the 300 included within the three matrices. The experimenter counted the numbers retrieved in a thirty seconds period and calculated the PI, i.e. the velocity of retrieval in numbers per second. The matrices presented in the different conditions analyses differed for the position of the target numbers, so that subjects could not benefit of previous spatial information for speeding up their performance.

### Assessment of the occlusion

**Fig 3:**
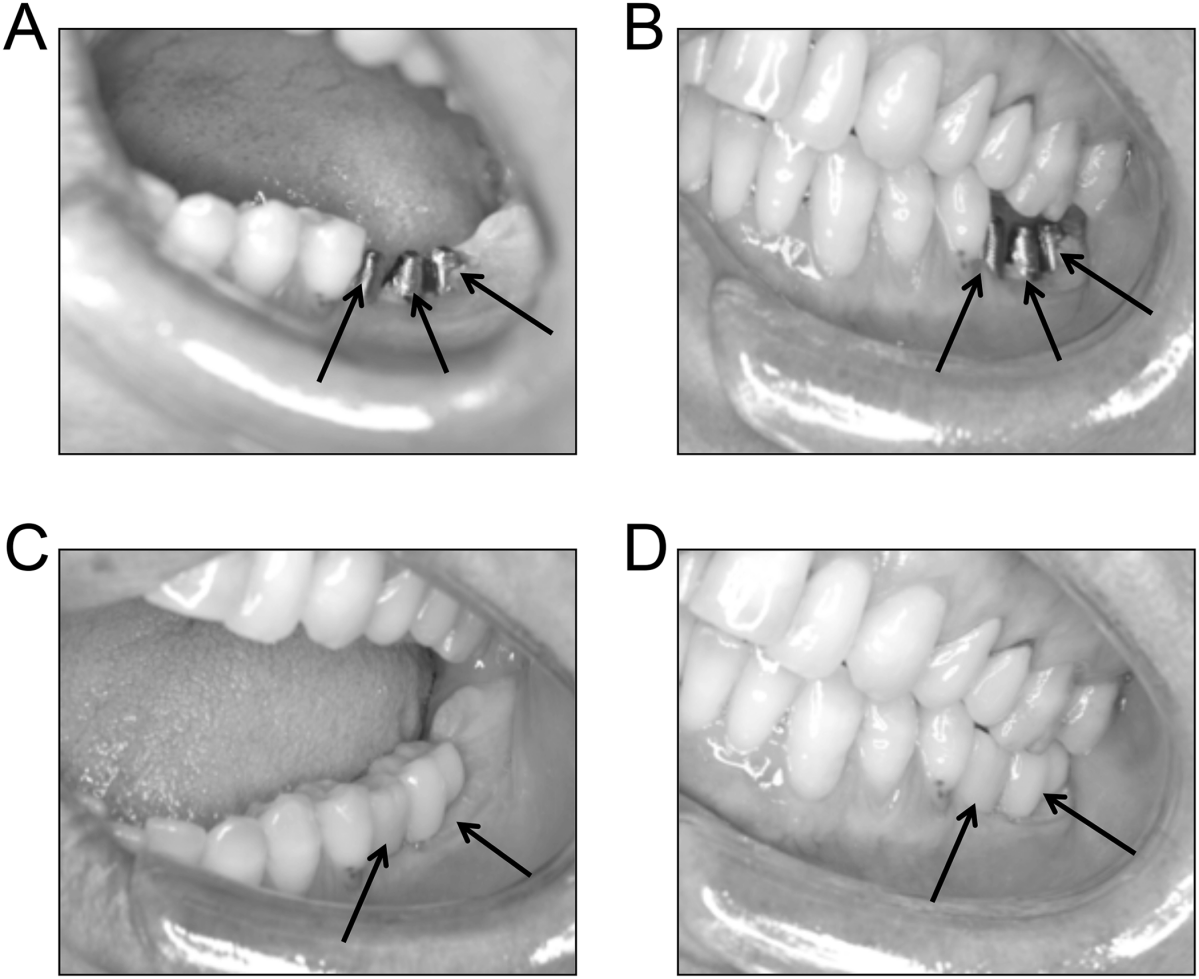
Evaluation of the intercuspal position. Camera shots of the arches of a subject, positioned apart (A, C) and touching each other (B, D). A-B: CROWN OFF. The arrows indicated the implants (n = 3). C-D: CROWN ON. The arrows indicated the prostheses (n = 2).

In five patients, the occlusal contact on the side of molar loss was assessed by camera shots at thirty days from the initial surgery, with and without placement of the prostheses. In this way it was possible to verify that the occlusion of the natural teeth were not modified by crown placement (see Fig 3).

### EMG recordings

**Fig 4:**
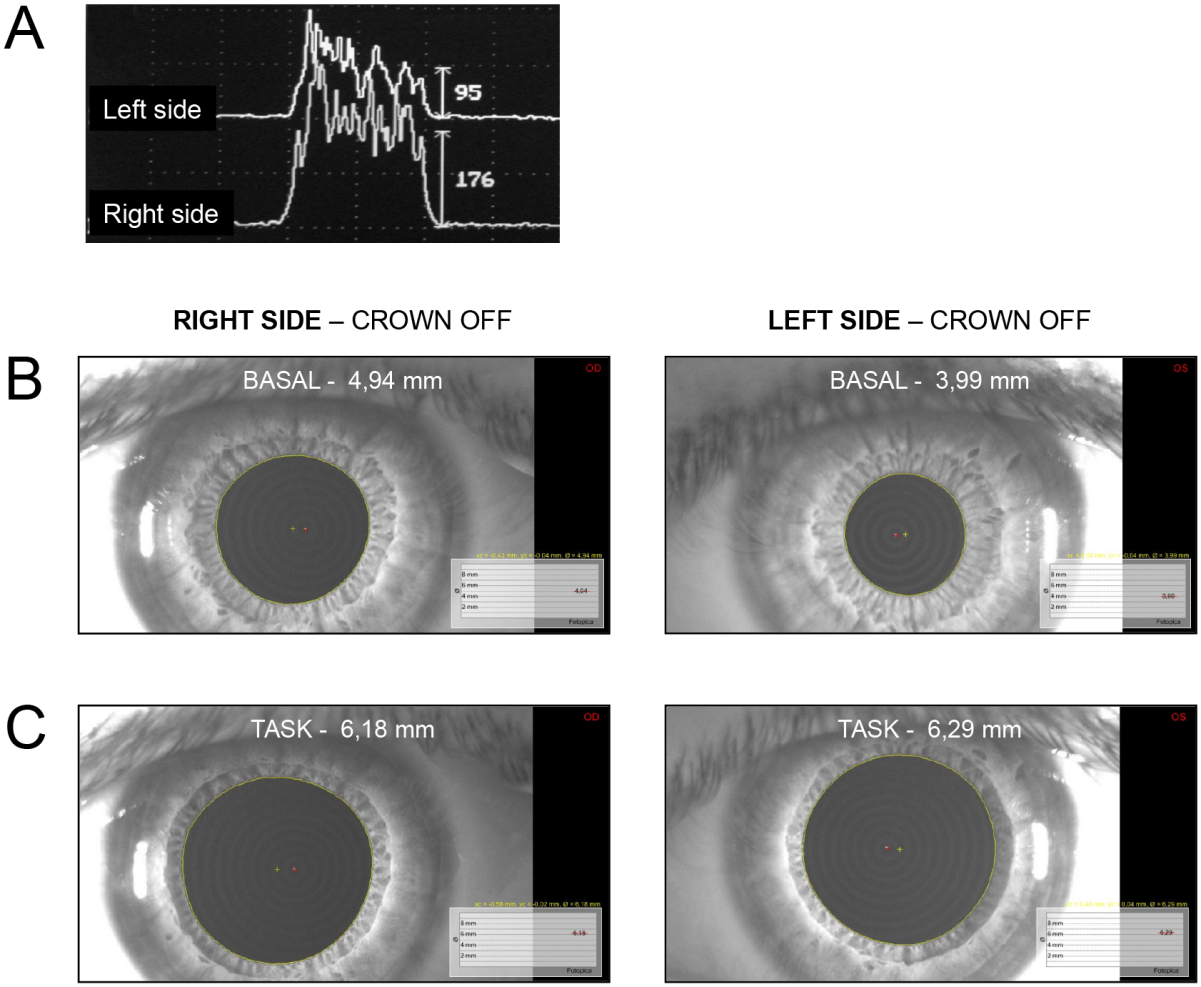
Examples of EMG and pupil size recordings. A. EMG activity recorded from left and right masseters in a given subject, without crown positioned and with the arches in contact (CROWN OFF). The vertical lines are calibration bars corresponding to the signal amplitude in μV. B. Recordings of pupil size in basal condition. Note that the left-right difference in pupil size is -0.95 mm. C. Recordings of pupil size during task performance (Tan Gram). Note that the left-right difference in pupil size was reduced to only 0.11 mm.

The EMG activity of masseter muscles was recorded by Duo-trode surface Ag/AgCl electrodes (interelectrode distance 19.5 mm, MyoTronics, Seattle, WA, USA). Electrodes were placed on the masseters belly, along an axis joining the orbit corner to the mandibular gonion, two cm far from the latter. The lead axis was parallel to the longitudinal axis of muscle fibres. Data were acquired at the sampling rate of 720 Hz by using an integrated system for EMG activity and mandibular movement recording (K6-I; MyoTronics). EMG signals were acquired with a lower cutoff frequency of 15 Hz, filtered with a notch (50 Hz), full-wave rectified and displayed on the instrument monitor. The instrument provided the mean value of the rectified EMG bursts produced during clenching. Recording was allowed by the instrument software only when the resistance of the two recording leads was comparable, which allows to minimize possible bias in the asymmetry evaluation due to the different size of the EMG signal of the two sides. During evaluations of EMG asymmetries subjects were asked to develop a strong clenching effort, which abruptly raised their EMG activity (Fig 4A). At this point they were ask to raise again the effort level, leading, in general to a further increase of the EMG signal. The total time of clenching effort ranged, in different subjects, from one to three seconds.

### Statistical analysis (SPSS.13)

First, in all patients we studied the left-right differences in pupil size at rest and during the haptic task as well as left-right differences in the masseter EMG activity during clenching. Positive and negative values indicated a left and right dominance, respectively. The correlation between pupil and EMG asymmetries was assessed by Pearson correlation coefficient.

The differences in size between the larger (mydriatic) and the smaller (miotic) pupil (pupil asymmety) and the PI, were submitted to a three condition (NO CONTACT, CROWN OFF, CROWN ON) repeated measures ANOVA. The difference in the mean value of the rectified EMG burst between the hyperactive and the hypoactive side (EMG asymmetry) was analysed in a two condition (CROWN OFF, CROWN ON) repeated measures ANOVA.

Secondly, we analysed the pupils size at rest and during haptic task and the corresponding task-rest difference (mydriasis) according to a 3 condition (NO CONTACT, CROWN OFF, CROWN ON) x 2 sides (miotic/mydriatic) repeated measures ANOVA.

The EMG activity was analysed according to a two conditions (CROWN OFF, CROWN ON) x two sides (miotic/midriatic) repeated measures ANOVA. In all instances, gender was a between subjects factor.

Third, in the five patients re-tested six months following the initial session, pupil size (rest and task) and mydriasis data were processed according to a 4 condition (NO CONTACT, CROWN OFF 1, CROWN ON, CROWN OFF 2) x 2 sides (miotic/mydriatic), repeated measures ANOVA. A 4 condition (NO CONTACT, CROWN OFF 1, CROWN ON, CROWN OFF 2) design was used for pupil asymmetry and PI. A three condition (CROWN OFF 1, CROWN ON, CROWN OFF 2) design was utilized for EMG asymmetries. In these analyses gender was not considered as a between subjects factor due to the small sampling size.

Finally, the repetition effect on PI was studied in 9 control subjects showing EMG and pupil size asymmetries, but no dental loss. Data were analysed according to a 3 times (T1, T2, T3) repeated measures ANOVA, without between subjects factor.

The Greenhause-Geisser ε correction was used when requested. Significance was set at p<0.05.

## Results

### Side differences in pupil size and activity

**Fig 5:**
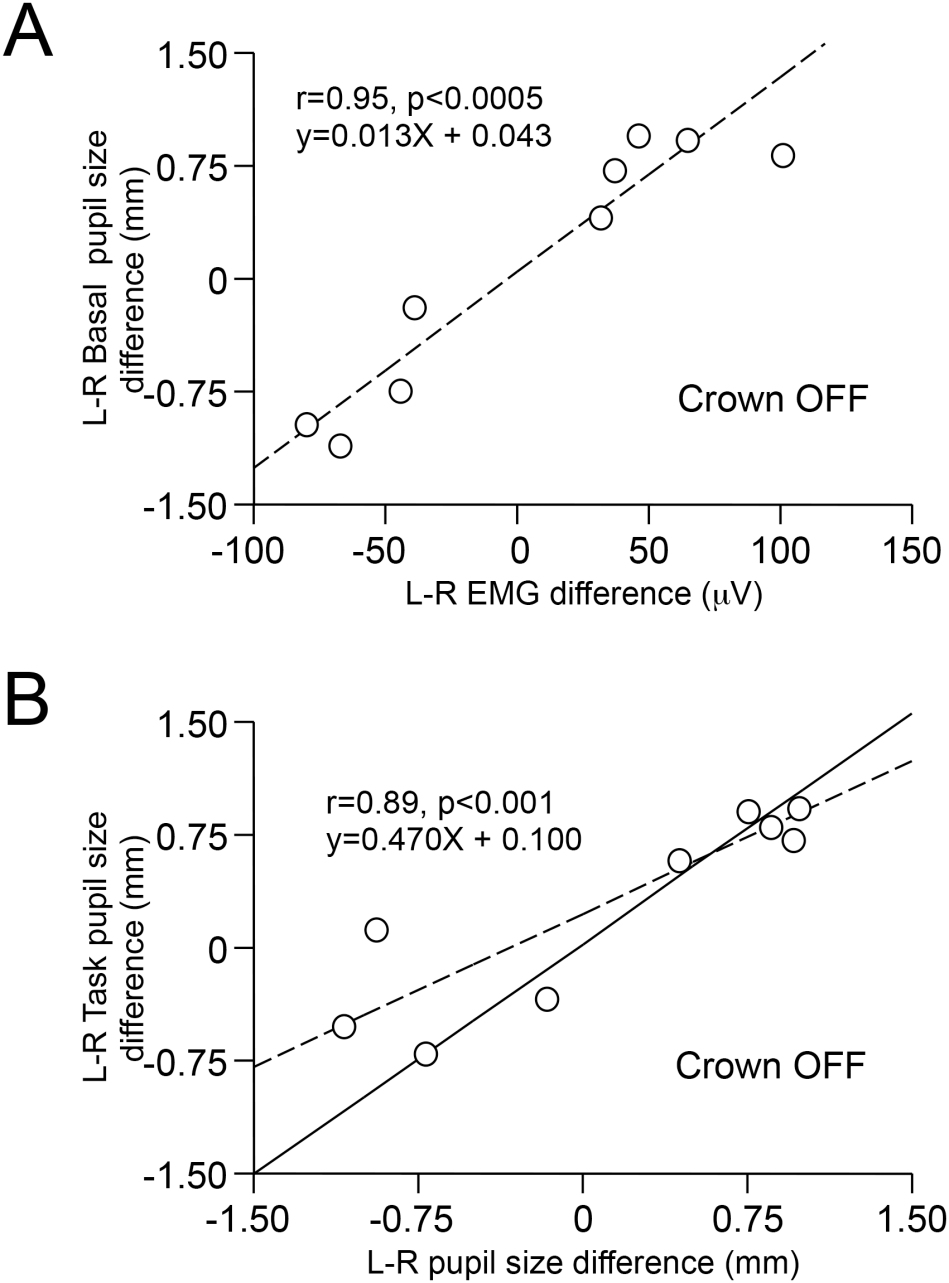
EMG and pupil’s size asymmetries in CROWN OFF condition. A. Relation between the left-right difference in pupil size measured with the arches in contact (CROWN OFF) and the subject not attending any task (Basal) and that in masseter EMG activity observed with the arches in contact (CROWN OFF) during bite. B. Relation observed in CROWN OFF between the left-right difference in pupil size measured during the haptic task and that observed in basal condition. The continuous line represents equal values of basal and task pupil’s asymmetries. In both A and B, dotted lines are the regression lines of the plotted points and their equations and coefficient of correlations are indicated in the insets.

All the subjects analysed in the CROWN OFF condition showed asymmetries in EMG activity of masseter muscles during biting (Fig 4A) and in the basal pupil size (Fig 4B). The side of the larger pupil always corresponded to the side of the higher EMG activity. Most often, the asymmetry in pupil size persisted when the subjects were involved in the haptic task. As shown in Fig 5A, a significant correlation existed between the left-right difference in basal pupil size and that in masseter EMG activity. A significant correlation was also observed between left-right pupil size differences in basal and task conditions (Fig 5B).

**Fig 6:**
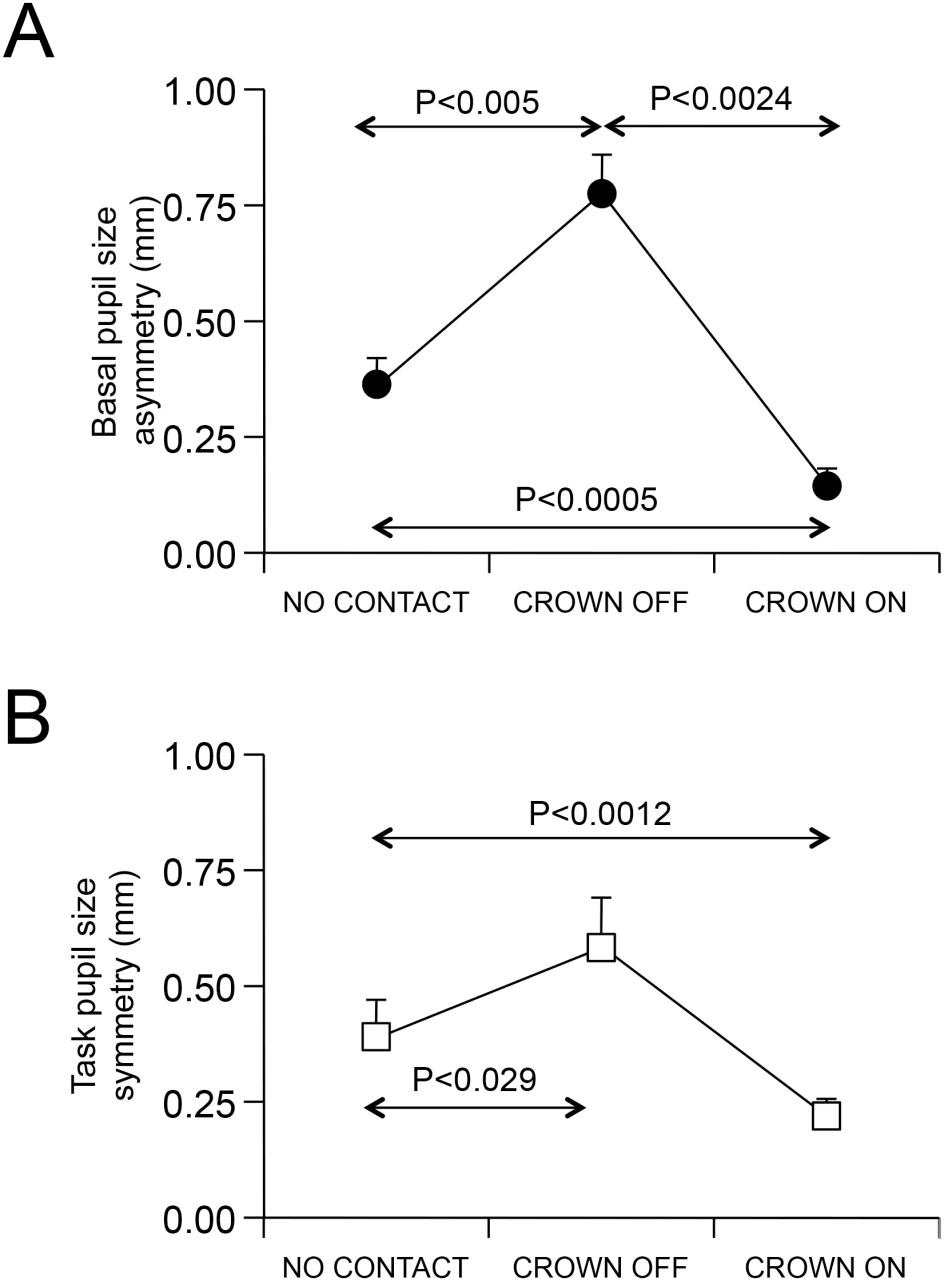
Pupil’s size asymmetries in different conditions. The average difference in pupil size between the larger and the smaller pupil has been displayed for each of the three conditions analysed. A: asymmetries recorded while the subjects were relaxed. B: asymmetries recorded during the performance of the haptic task. In both A and B, the error bars represent standard deviations.

Crown placement greatly reduced (F(1,7) = 42.71, P<0.0005) the EMG asymmetry between the mydriatic (higher EMG activity) and the miotic side (lower EMG activity), which dropped from 56.67 ± 23.15, SD, μV (CROWN OFF) to 13.11 ± 10.08, SD, μV (CROWN ON). A similar result (ANOVA: (F(2,14) = 45.19, p<0.0005), was obtained for basal pupils asymmetry, which increased from NO CONTACT to CROWN OFF condition and dropped to the lowest value in CROWN ON condition (see Fig 6A). No significant gender effects were observed.

**Fig 7:**
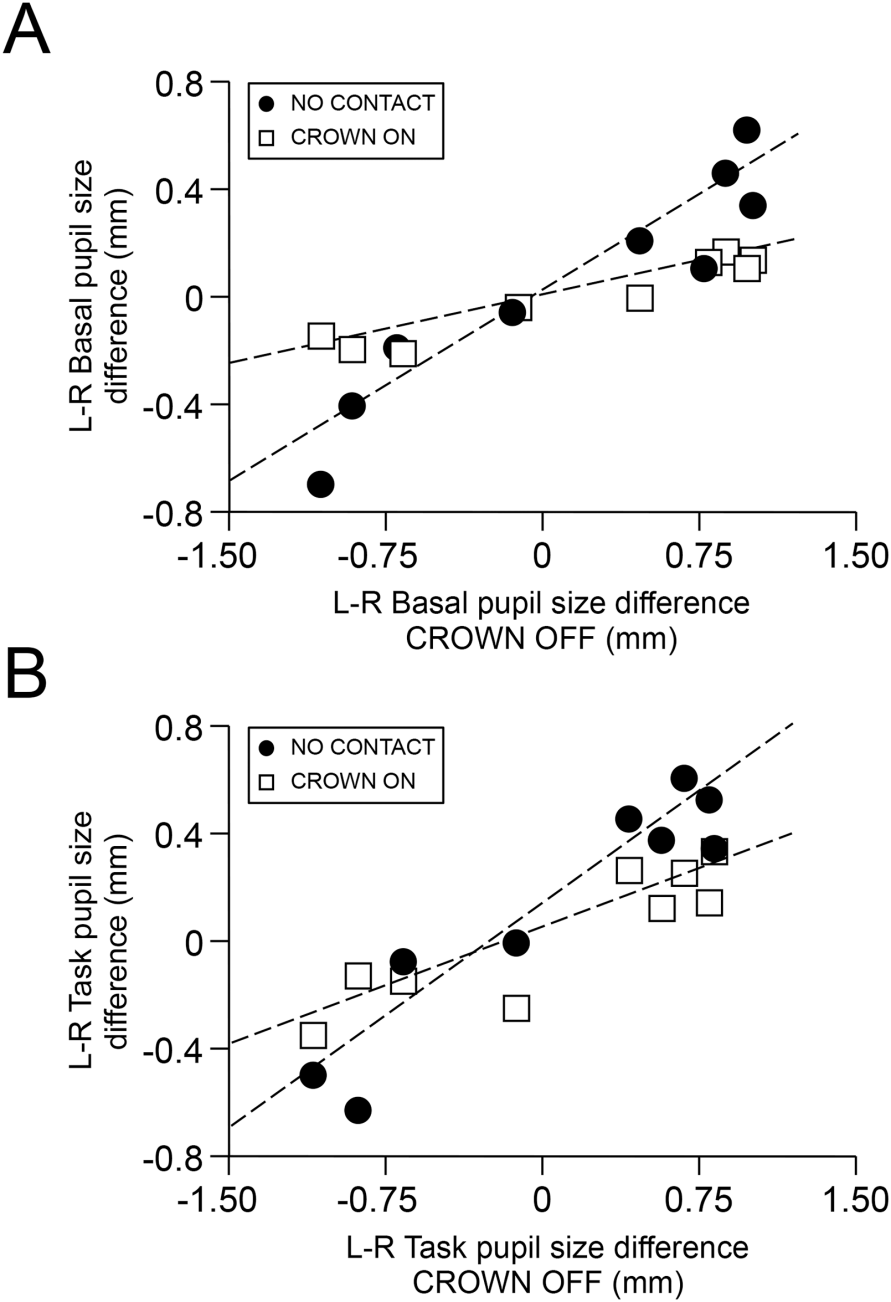
Relations between left-right pupil size difference in different conditions. Dots and open squares represent the left-right pupil size differences recorded in NO CONTACT and CROWN ON condition respectively, plotted as a function on the corresponding values obtained in the CROWN OFF condition. A. Basal pupil size asymmetry. B. Task pupil size asymmetry. Dotted lines are the regression lines obtained for dots and open square data, which correspond to the following equations: A. dots: r = 0.941, P<0.0005, Y = 0.468X + 0.18; squares: r = 0.958, P< 0.005, Y = 0.167X + 0.001. B. dots: r = 0.945, P<0.0005, Y = 0.666X–0.027; squares: r = 0.8878, P< 0.001, Y = 0.345X–0.036.

Similar results could be also obtained for pupil asymmetry during the haptic task (F(2,14) = 8.30, P<0.004). Nonetheless, in this instance, the difference between the NO CONTACT and CROWN ON conditions was not significant (see Fig 6B). As shown in Fig 7, the left-right differences in basal and task pupil size observed in NO CONTACT and CROWN ON were strongly correlated to those observed in CROWN OFF. The same occurred for EMG differences observed in CROWN ON and CROWN OFF (r = 0.88, P<0.002, Y = 0.225X + 3.84).

### Performance Index

**Fig 8:**
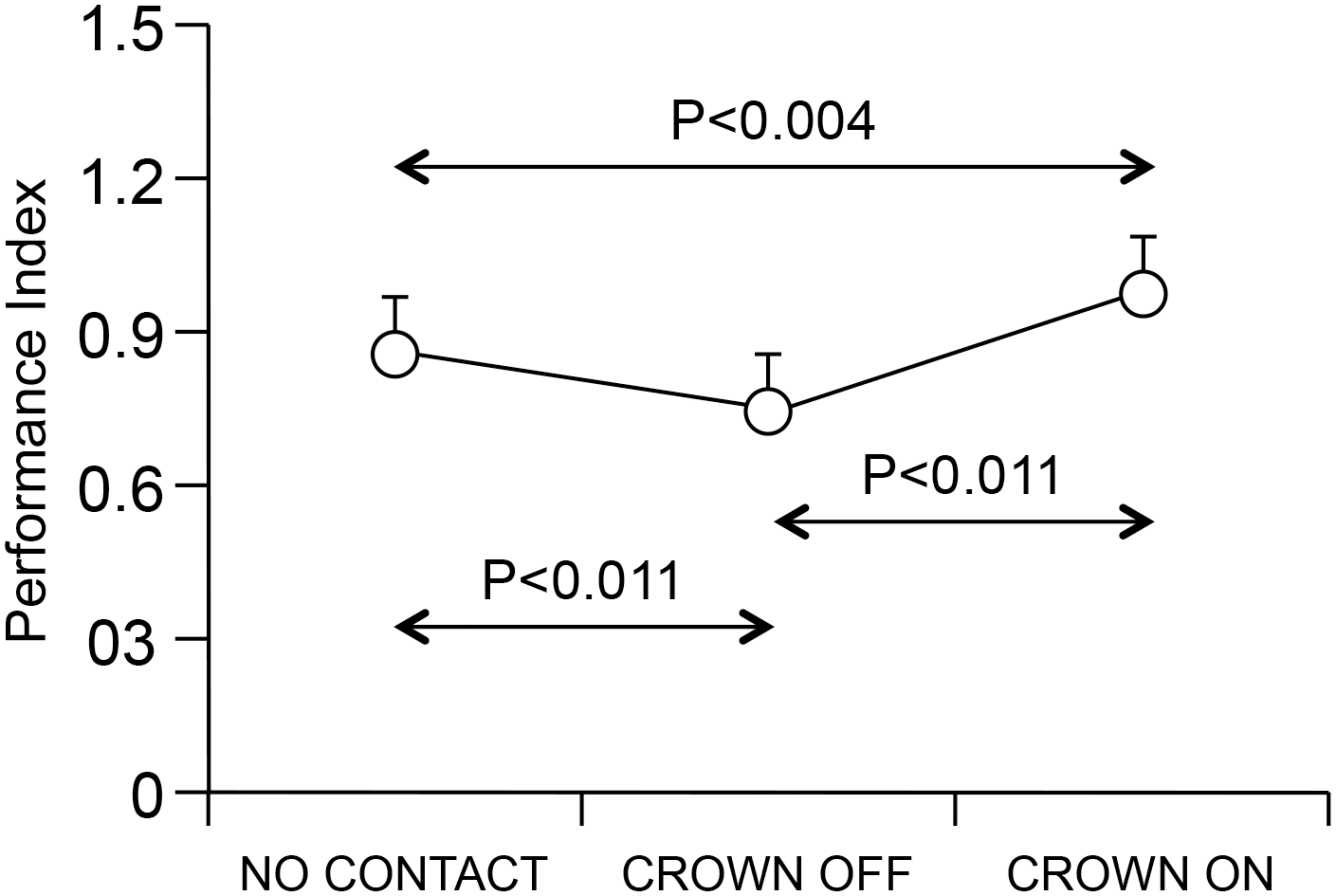
Performance Index in different conditions. The average values of PI have been displayed for each of the three conditions analyzed. The error bars represent standard deviations.

The significant condition effect observed for the PI (F(2,14) = 248.57, P<0.0005), indicated that this parameter decreased from NO CONTACT to CROWN OFF condition, while increased above the NO CONTACT values in CROWN ON. As shown in Fig 8, post-hoc comparison indicated that each condition differed significantly from the others.

### Pupil size, EMG activity and mydriasis on the two sides

**Fig 9:**
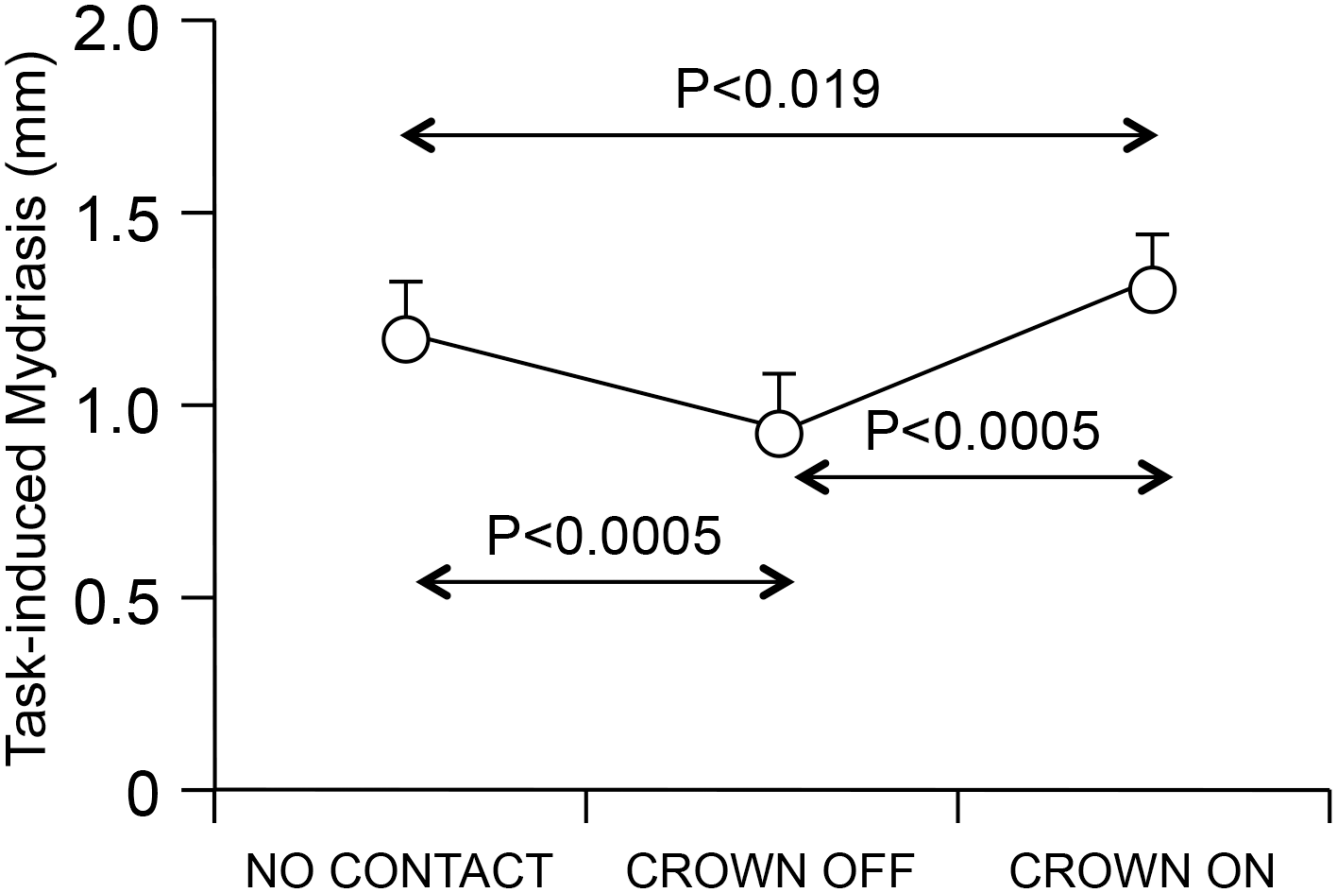
Task-induced mydriasis in different conditions. The average values of the task induced mydriasis have been displayed for each of the three conditions analyzed. The error bars represent standard deviations. For each subject, values relative to the left and right pupils were averaged.

Significant results are reported in Table 1. Table 2 reports mean ± SD values of EMG and pupil size. These data clarify the origin of the changes in EMG and pupils asymmetries previously described. Significant condition, side and condition x side effects were found for both basal and task pupil size. Side effects were due to the fact that 1) basal sizes of larger pupils were pooled together and compared to those of the smaller ones and 2) task pupil size was in general larger in the pupil with larger basal size. Decomposition of the significant interaction (see Table 2) revealed that basal size in NO CONTACT and CROWN OFF was similar in the smaller pupil; in contrast, in the larger one, NO CONTACT values were smaller then CROWN OFF. Thus it appears that closing the arches without crowns increases the asymmetry in basal pupil size. On the other hand, basal size of the smaller pupil increased in CROWN ON with respect to CROWN OFF, while that of the larger pupil decreased in CROWN ON: this explains why the asymmetry decreased in CROWN ON with respect to CROWN OFF. On the other hand, task size of both the smaller and larger pupils did not significantly differed between NO CONTACT and CROWN OFF. Nonetheless a slight decrease in the smaller and an increase in the larger pupil size when changing from NO CONTACT to CROWN OFF led to an enhancement of the asymmetry in CROWN OFF with respect to NO CONTACT. Finally, both pupils increased their task size in CROWN OFF with respect to CROWN ON. Since the increase was larger in the smaller pupil, the left-right side asymmetry decreased in CROWN ON. Modifications in basal and task pupil size modified the task-related mydriasis, which decreased from NO CONTACT to CROWN OFF and increased in CROWN ON (see Fig 9), with more marked changes in the larger with respect to the smaller pupil (see Table 2). As to the EMG activity, it has to be pointed out that the hypoactive muscle showed a significant increase in CRONW OFF with respect to CROWN ON, whereas the hyperactive one did not change.

**Table 1:** Statistically significant effects and interactions observed in the first experimental session.

Variable	Effect	p <	η^2^	Post Hoc (T-Test)	p <	T
**Pupil Size (Basal)**	Condition F(2,14) = 14.49	0.0005	0.674	NO CONTACT < CROWN OFF	0.0005	-7.116
				CROWN ON > NO CONTACT	0.006	-3.691
	Side F(1,7) = 57.16	0.0005	0.891	miotic < mydriatic	0.0005	-7.043
	Condition x Side F(2,14) = 45.19	0.0005	0.866	Decomposed in Table 2		
**Pupil Size (Task)**	Condition F(2,14) = 24.78	0.0005	0.780	CROWN OFF < CROWN ON	0.0005	-7.815
				CROWN ON > NO CONTACT	0.001	-5.038
	Side F(1,7) = 37.41	0.0005	0.842	miotic < mydriatic	0.0005	-6.559
	Condition x Side F(2,14) = 8.30	0.004	0.543	Decomposed in Table 2		
**Mydriasis**	Condition F(2,14) = 46.73	0.0005	0.870	NO CONTACT > CROWN OFF	0.0005	8.897
				CROWN OFF < CROWN ON	0.0005	-8.458
				CROWN ON > NO CONTACT	0.013	-3.174
	Side F(1,7) = 0.000	0.987	0.000			
	Condition x Side F(2,14) = 3.90	0.045	0.357	Decomposed in Table 2		
**EMG Activity**	Condition F(1,7) = 5.14	0.058	0.423	CROWN OFF<CROWN ON	0.045	-2.379
	Side F(1,7) = 52.91	0.0005	0.883	miotic < mydriatic	0.0005	-7.096
	Condition x Side F(1,7) = 42.71	0.0005	0.859	Decomposed in Table 2		

Statistical significant effects observed in a three condition (NO CONTACT, CROWN OFF, CROWN ON) x 2 sides (miotic, mydriatic) repeated measures ANOVA performed on 9 patients. See text for further explanations.

**Table 2:** Mean values of EMG and pupil size observed in the first experimental session.

		Conditions					
		1. NO CONTACT		2. CROWN OFF		3. CROWN ON	
Side	Variabiles	mean±SD	post-hoc 1–2	mean±SD	post-hoc 2–3	mean±SD	post-hoc 3–1
**Smaller pupil (miotic)**	basal size	3.31±0.43	NS	3.37±0.36	P<0.011	3.62±0.46	P<0.0005
	task size	4.47±0.82	NS	4.39±0.83	P<0.0005	4.89±0.67	P<0.002
	mydriasis	1.16±0.42	P<0.061	1.02±0.52	P<0.024	1.26±0.31	NS
	EMG (hypoactive)			86.8±35.4	P<0.005	130.0±54.5	
**Larger pupil (mydriatic)**	basal size	3.65±0.60	P<0.0005	4.12±0.54	P<0.0005	3.75±0.49	NC
	task size	4.86±0.66	NS	4.98±0.66	P<0.010	5.11±0.68	P<0.003
	mydriasis	1.21±0.33	P<0.0005	0.85±0.28	P<0.0005	1.36±0.33	P<0.011
	EMG (Hyperactive)			143.5±50.5	NS	143.1±52.5	

Average ± standard deviation values of the variables recorded in the nine subjects analized, submitted to a 3 condition x 2 sides repeated measures ANOVA. The EMG activity recorded on the side of the larger pupil was always higher than that recorded on the opposite side. P values refer to post-hoc analysis (see Methods for further explanations).

### Effects of test repetition in patients

In order to evaluate the effect of test repetition, five of the tested subjects with unilateral molar loss were studied once more, at six months from the initial session. In this second session, an additional final test in CROWN OFF was performed. Analysis of these data revealed condition-related changes in EMG, pupil size and mydriasis comparable to those documented in the first session (see Tables 3 and 4), although some differences were not significant, likely owing to the smaller number of subjects. Repetition of the condition did not change the EMG and pupil diameter, as CROWN OFF 1 and 2 were very similar. A significant condition effects (F(3,12) = 55.02, P<0.0005) was observed for the PI and post hoc analysis revealed smaller values in Crown OFF 1 (0.76 ± 0.27, P<0.001) and CROWN OFF 2 (0.75 ± 0.27, P<0,002) with respect to CROWN ON (0.96 ± 0.25. In contrast, no significant difference could be found between CROWN OFF 1 and 2. On the other hand the PI value observed in the NO CONTACT condition (0.87 ± 0.29) was significantly higher with respect to CROWN OFF (1: P<0.001, 2: P<0.001) and lower with respect to CROWN ON (P<0.016).

**Table 3:** Statistically significant effects and interactions observed in the second experimental session.

Variable	Effect	p <	η^2^	Post Hoc (T-Test)	p <	t
**Pupil Size (Basal)**	Condition F(3,12) = 4.82	0.055	0.546	NO CONTACT < CROWN OFF1	0.013	-4.218
				NO CONTACT < CROWN OFF2	0.019	-3.820
	Side F(1,4) = 21.14	0.010	0.841	miotic < mydriatic	0.010	4.597
	Condition x Side F(3,12) = 27.09	0.004	0.871	Decomposed in Table 4		
**Pupil Size (Task)**	Condition F(3,12) = 13.28	0.0005	0.768	CROWN OFF1<CROWN ON	0.007	-5.069
				CROWN ON>NO CONTACT	0.032	-3.231
				CROW OFF2<CROWN ON	0.008	4.919
	Side F(1,4) = 9.34	0.038	0.700	miotic < mydriatic	0.038	3.055
	Condition x Side F(3,12) = 5.80	0.066	0.592	Decomposed in Table 4		
**Mydriasis**	Condition F(3,12) = 12.54	0.009	0.758	NO CONTACT > CROWN OFF1	0.039	3.030
				CROWN OFF1 < CROWN ON	0.005	-5.654
				CROW OFF2 < CROWN ON	0.004	6.101
	Side F(1,4) = 0.22	0.664	0.052			
	Condition x Side F(3,12) = 1.87	0.240	0.319	Decomposed in Table 4		
**EMG Activity**	Condition F(2,8) = 11.94	0.024	0.749	CROWN OFF1 < CROWN ON	0.019	-3.816
				CROWN OFF2 < CROWN ON	0.034	3.174
	Side F(1,4) = 152.86	0.0005	0.974	miotic < mydriatic	0.0005	12.363
	Condition x Side F(2,8) = 7.40	0.050	0.649	Decomposed in Table 4		

Statistical significant effects observed in a four condition (NO CONTACT, CROWN OFF 1, CROWN ON, CROWN OFF 2) x 2 sides (miotic/mydriatic) repeated measures ANOVA performed on 5 of the 9 patients illustrated in Table 1. See text for further explanations.

**Table 4:** Mean values of EMG and pupil size observed in the second experimental session.

		Conditions									
		1. NO CONTACT			2. CROWN OFF (1)			3. CROWN ON			4. CROWN OFF (2)
Side	Variables	mean±SD	post-hoc 1–2	post-hoc 1–4	mean±SD	post-hoc 2–3	post-hoc 2–4	mean±SD	post-hoc 3–1	post-hoc 3–4	mean±SD
**Smaller pupil (miotic)**	basal size	3.36±0.63	NS	NS	3.31±0.49	P<0.055	NS	3.60±0.49	NS	P<0.04	3.30±0.48
	Task size	4.73±0.99	NS	NS	4.51±1.07	P<0.008	NS	5.03±0.84	P<0.046	P<0.017	4.54±1.09
	Mydriasis	1.37±0.40	NS	NS	1.20±0.62	NS	NS	1.43±0,36	NS	NS	1.24±0.64
	EMG (hypoactive)				75±32.95	P<0.021	NS	133±62.45		P<0.034	77.4±31.13
**Larger pupil (mydriatic)**	basal size	3.45±0.63	P<0.002	P<0.003	3.93±0.65	P<0.039	NS	3.64±0.54	NS	P<0.038	3.95±0.65
	Task size	4.88±0.94	NS	NS	4.98±0.88	NS	NS	5.15±0.87	P<0.022	NS	4.98±0.85
	Mydriasis	1.43±0.35	P<0.011	P<0.014	1.05±0.26	P<0.006	NS	1.51±0.37	NS	P<0.005	1.03±0.24
	EMG (hypoactive)				116±42.10	P<0.022	NS	141.80±51.42		P<0.064	120.4±44.78

Average ± standard deviation values of the variables recorded in the five of the nine subjects shown in Table 2, s submitted to a 4 condition x 2 sides repeated measures ANOVA. The EMG activity recorded on the side of the larger pupil was always higher than that recorded on the opposite side. P values refer to post-hoc analysis (see Methods for further explanations).

### Effects of test repetition in normal subjects

Control subjects showed a significant correlation between left-right pupil size (at rest and during task) and EMG differences (basal: r = 0.802, P<0.0005, Y = 0.007X -0.006; task: r = 0.893, P<0.001, Y = 0.006X + 0.078). The left-right pupil size asymmetry observed during task correlated with the corresponding value at rest (r = 0.306, P<0.424, Y = -0.257X + 0.168). No significant time effect could be found for the PI (mean values: T1, 0.89 ± 0.29; T2, 0.92 ± 0.33; T3, 0.91 ± 0.32).

## Discussion

The present findings indicate that, in subjects deprived unilaterally of the first and second molar, an asymmetric EMG activity of masticatory muscles develops when the arches are in contact. The side difference in EMG activity is highly correlated with the side difference observed in pupil size; both variables are smaller on the implant side. The same has been previously observed in subjects affected by TMD [26], showing asymmetry of masseter EMG activity during bite.

The observed asymmetry in pupil size was rather small, ranging from 0.18 to 0.95 mm in the different subjects (CROWN OFF, basal condition). However, it was still present, together with the correlated EMG asymmetry, six months later, which suggests it is a stable trait of subjects with unbalanced activity of elevator muscles.

In the present study, the observed EMG unbalance of masticatory muscles could depend on side differences in trigeminal sensory signals elicited during biting, which could induce asymmetric activity of trigeminal motor nuclei. In this respect it has to be pointed out that periodontal mechanoreceptors (PMRs) supply information about the forces applied to the teeth and contribute to the regulation of muscle activity generating masticatory and jaw movements [33,34]. The activation of low threshold PMRs, by the pressure exerted on the teeth can excite at short latency masseter α and γ motoneurons [34]. So, unilateral molar loss, which reduces PMRs inputs, can contribute to the ipsilateral depression of masseter EMG activity. It is known that higher threshold PMRs give rise to the opposite effect, facilitating jaw opening [35]. This reflex component is reduced during the closing phase of chewing [36] and it is likely that the same phenomenon occurs during voluntary clenching. Crown placement did not modify the EMG activity on the hyperactive side, whereas it enhanced hypoactive side activity, possibly due to recruitment of the PMRs of the upper arch.

Another possible explanation for EMG asymmetry could be that lack of crowns on one side modifies the position of the mandible, inducing side differences in the sarcomere length of masseters and, as a consequence, in force development and motor units activity during clenching. Nonetheless, no obvious differences in the intercuspal position of patient’s natural teeth could be observed when the arches were in contact with and without implanted crowns, provided that the occlusal level had been refined by milling the crown surface.

In the present study molar teeth loss-induced malocclusion seems to be the cause of the masticatory asymmetry, as the latter disappeared after crown placement. On the other hand, in some patients, malocclusion could be the consequence of prolonged, asymmetric masticatory efforts of central origin, rather than its cause. A central contribution to the masticatory asymmetry could explain why a couple of our patients showed residual EMG asymmetry following crown placement. Further investigations are required to disentangle the central and peripheral components of the muscles asymmetric activity.

As previously observed [26], the correction of EMG asymmetry was associated to a correction of pupil asymmetry. This highlights the influence of asymmetric trigeminal information on structures controlling the pupils diameter, thus emphasizing the dependence of the latter upon a trigeminal sensorimotor unbalance. The asymmetry in pupil size, which was lower when arches were in rest position (teeth 1–2 mm apart), owing to the scarce information rising from PMRs [33] and its increase by dental contact is in line with this view.

Trigeminal control of autonomic structures may develops through different pathways. Although trigeminal afferent fibres have no access to the ciliary and superior cervical ganglion [37], trigeminal input has been shown to increase the discharge of superior cervical ganglion neurons [38]. Trigeminal afferents reach autonomic structures, such as the nucleus of tractus solitarius, the ventrolateral medulla, the A5 area, the ventrolateral part of the parabrachial nucleus and the Kolliker-Fuse nucleus [39]. In addition, they reach the parvicellular reticular formation [40, 41], which mediates autonomic reflexes [42] and may influence the preganglionic parasympathetic neurons located within the Edinger-Westphal nucleus through the reticular formation and the vestibular nuclei [43,44,45]. The most important pathway is likely passing through Locus Coeruleus (LC neurons), which respond to transcutaneous electrical stimulation of the hamster’s pinna [46], receive afferents from neurons localized within or near to the trigeminal Mesencephalic nucleus [47] and are electrically coupled to proprioceptive trigeminal afferents [48]. Moreover, noradrenergic LC neurons projects to the preganglionic parasympathetic neurons of the Edinger-Westphal nucleus [43] and inhibits their discharge [49,50]. This inhibition is necessary to increase the pupil size, since the tonic activity of the iris constrictor would prevent pupil enlargement by dilatator pupillae [51]. The LC discharge and pupil size covary both in animals [31,52] and humans [53] and, indeed, pupil size is now considered as an indicator of LC activity [54,55,56]

Asymmetric trigeminal input to LC could be at the basis of pupils asymmetry. This hypothesis is consistent with the fact that occlusal disharmony increased the release of noradrenaline in the hypothalamic paraventricular nucleus [57] and in the frontal cortex [58].

Crown placement on dental implants also enhanced the mydriasis associated with a haptic task. Mydriasis is strictly proportional to the parallel task-related (phasic) release of noradrenaline at cerebral cortical level [59]. Such release originates from the activation of LC, which modulates cortical arousal [50,60,61]. In contrast, LC high tonic activity reduces the phasic release of noradrenaline, decreasing task-performance [62]. So the present data suggest that reduction of the sensorimotor asymmetry by teeth substitution can reduce the asymmetry in LC discharge of the two sides and also increase the LC phasic activation, leading to an enhancement of the mydriasis associated with a cognitive task.

It is noteworthy that noradrenaline controls intracellular (Ca^2+^) inflow in astrocytes [63], which play a key role in regulating cerebral blood flow [63,64,65], astrocytic glucose metabolism [66] as well as the cerebral synthesis and the release of BDNF [67], which is critical for long-term potentiation [68] and spatial memory [69]. This mechanism may be involved in the LC-noradrenaline system control of cognitive [70].

Thus, the improvement of the performance at the Spinnler-Tognoni matrices task induced by crown placement could be the consequence of a better phasic activation of LC neurons. Such improvement cannot be attributable to mere task repetition of the test, as the latter did not modify the PI in control subjects and the improvement elicited by crown placing in patients was completely abolished by its successive removal.

Cognitive performance may also be improved by the reduction in the left-right asymmetry of the LC tonic discharge, which may lead to a different excitability of the two hemispheres and, as a consequence, to a deterioration of cognitive performance. It has been shown, in fact, that lesion-induced unbalance in hemispheric activity may lead to severe cognitive deficits which disappears after a second, symmetric lesion on the opposite side, which doubles the extension of brain damage [71].

So, trigeminal input to the LC and, possibly, to other regions of the Ascending Reticular Activating System may help to regulate brain excitability. This hypothesis is consistent with the observation that trigeminal stimulation is useful for symptoms relief in epilepsy [72,73,74] and depression [75,76].

In this respect it has to be pointed out that also vagal stimulation may exert important effects on brain functions. In fact, vagal nerve stimulation is an approved treatment for epilepsy, depression and Alzheimer Disease [62,63] and these effects have been attributed to an activation of LC and the raphe nucleus.

Finally, it is noticeable that the masticatory activity enhances the production of BDNF and Neurotrophine-3 by muscle tissue, which are important neurotrophic factors for LC neurons and their axons [77] and this process could co-operate with the short-term influence of trigeminal afferents on LC. Thus masticatory dysfunction could impair LC neuronal functions and trigger long-term neurodegenerative processes.

In conclusion, our finding suggests that:

1. trigeminal unbalance induces asymmetric LC discharge which may exert short and long-term influences on brain excitability, leading to an asymmetric pupils size and to cognitive deficits;
2. rebalancing the activity of trigeminal afferents not only improves the masticatory activity, but also makes pupil size pupils size symmetric and improves cognitive functions.
